# Influence of internalin a murinisation on host resistance to orally acquired listeriosis in mice

**DOI:** 10.1186/1471-2180-13-90

**Published:** 2013-04-23

**Authors:** Silke Bergmann, Philippa M Beard, Bastian Pasche, Stefan Lienenklaus, Siegfried Weiss, Cormac G M Gahan, Klaus Schughart, Andreas Lengeling

**Affiliations:** 1 Department of Infection Genetics, Helmholtz Centre for Infection Research & University of Veterinary Medicine Hannover, Braunschweig D-38124, Germany; 2 Infection and Immunity Division, The Roslin Institute and R(D)SVS, University of Edinburgh, Easter Bush Veterinary Campus, Edinburgh EH25 9RG, UK; 3 Molecular Immunology, Helmholtz Centre for Infection Research, Braunschweig D-38124, Germany; 4 Department of Microbiology and School of Pharmacy, Alimentary Pharmabiotic Centre, University College Cork, Cork, Ireland; 5 University of Tennessee Health Science Center, Memphis, TN, USA

## Abstract

**Background:**

The bacterial surface protein internalin (InlA) is a major virulence factor of the food-born pathogen *Listeria monocytogenes*. It plays a critical role in the bacteria crossing the host intestinal barrier by a species-specific interaction with the cell adhesion molecule E-cadherin. In mice, the interaction of InlA with murine E-cadherin is impaired due to sequence-specific binding incompatibilities. We have previously used the approach of ‘murinisation’ to establish an oral listeriosis infection model in mice by exchanging two amino acid residues in InlA. This dramatically increases binding to mouse E-cadherin. In the present study, we have used bioluminescent murinised and non-murinised *Listeria* strains to examine the spatiotemporal dissemination of *Listeria* in four diverse mouse genetic backgrounds after oral inoculation.

**Results:**

The murinised *Listeria monocytogenes* strain showed enhanced invasiveness and induced more severe infections in all four investigated mouse inbred strains compared to the non-murinised *Listeria* strain. We identified C57BL/6J mice as being most resistant to orally acquired listeriosis whereas C3HeB/FeJ, A/J and BALB/cJ mice were found to be most susceptible to infection. This was reflected in faster kinetics of *Listeria* dissemination, higher bacterial loads in internal organs, and elevated serum levels of IL-6, IFN-γ, TNF-α and CCL2 in the susceptible strains as compared to the resistant C57BL/6J strain. Importantly, murinisation of InlA did not cause enhanced invasion of *Listeria monocytogenes* into the brain.

**Conclusion:**

Murinised *Listeria* are able to efficiently cross the intestinal barrier in mice from diverse genetic backgrounds. However, expression of murinized InlA does not enhance listerial brain invasion suggesting that crossing of the blood brain barrier and crossing of the intestinal epithelium are achieved by *Listeria monocytogenes* through different molecular mechanisms.

## Background

*Listeria monocytogenes* is a Gram-positive, facultative intracellular pathogen that can infect humans and animals after ingestion of contaminated food. It is responsible for human listeriosis, a disease predominantly affecting immunocompromised individuals. It can manifest itself in a wide range of clinical symptoms including meningitis or meningoencephalitis, gastroenteritis, abortion, perinatal infection, and septicemia [1,2]. Central to the pathogenesis of listeriosis is the ability of the bacterium to cross host epithelial barriers. After oral infection *L. monocytogenes* can breach the intestinal barrier via invasion of intestinal epithelial cells or via transcytosis of goblet cells [3] or microfold (M) cells in Peyer’s Patches [4,5]. The pathogen is then able to spread systemically by the hematogenous and lymphatic route to internal organs. The ability of *L. monocytogenes* to cross the blood–brain and placental barriers to invade the central nervous system and the fetalplacental unit is associated with the most severe and often fatal forms of *Listeria* infections in immunocompromised patients and pregnant women [6].

Two bacterial surface proteins, Internalin A (InlA) and Internalin B (InlB) play a major role in the internalisation of *L. monocytogenes* into non-phagocytic cells and in the crossing of epithelial barriers [3,7-9]. The molecular interaction of both internalins with their respective receptors is species-specific. InlA induces listerial internalisation into intestinal epithelial cells by binding to the N-terminal domain of the human E-cadherin (Cdh1) cell adhesion protein [10]. It can also interact with Cdh1 from guinea pig, rabbit and gerbil but fails to bind to the corresponding domain of the murine and rat Cdh1. This species specificity is mostly determined by the presence of a proline at the 16th amino acid position of Cdh1 in permissive species and of a glutamic acid in non-permissive species [10-12]. InlB binds to the mouse, human, and gerbil Met receptor and can induce listerial uptake in a wide range of different mammalian cell types including hepatocytes and epithelial cells but cannot recognise the guinea pig and rabbit Met receptors [13,14]. The species-specific receptor interactions of InlA and InlB have limited the development of small animal models to study mechanisms of *L. monocytogenes* dissemination and pathogenesis after oral infection. A major breakthrough was the generation of a transgenic mouse line which expresses the human *E-cadherin* (*CDH1*) gene under the control of the enterocyte specific promoter of intestinal fatty-acid-binding protein. This mouse model demonstrated for the first time that the interaction of InlA with Cdh1 is crucial for listerial intestinal invasion *in vivo*[15]. More recently, a mouse E16P knockin model in which the glutamic acid at position 16 of the murine *Cdh1* gene was replaced by a proline was used to show that InlA and InlB are synergistically required for the crossing of the pathogen through the placental barrier after oral inoculation [16]. The ubiquitous expression of a ‘humanized’ Cdh1 in this mouse allows the investigation of InlA-Cdh1 and InlB-Met interactions *in vivo*.

We have previously taken a different route to generate an InlA and InlB permissive *L. monocytogenes* mouse infection model through an approach we call pathogen ‘murinisation’ [12]. Based on structural information on the recognition complex of InlA with the N-terminal domain of Cdh1, two amino acids in InlA were replaced (Ser192Asn and Tyr369Ser), dramatically increasing the binding affinity of murine Cdh1 to InlA [12]. By introducing these two mutations into the listerial *inlA* locus, a variant strain of *L. monocytogenes* EGD-e (Lmo-InlA^m^) was generated which was able to cross the murine intestinal barrier and to induce symptoms of listeriosis after oral inoculation [12]. In contrast to the *Cdh1* transgenic mouse models, this mouse model permits the analysis of orally acquired listeriosis without the need to cross in ‘humanized’ alleles of *Cdh1*.

In this study, we have employed a previously generated bioluminescent *L. monocytogenes* EGD-e strain (Lmo-InlA-mur-lux) ‘murinised’ for the two Ser192Asn and Tyr369Ser *inlA* mutations [17] and a ‘non-murinised’, isogenic control strain (Lmo-EGD-lux) to analyse host responses after oral infection in four different inbred strains of mice. C3HeB/FeJ, A/J, BALB/cJ, and C57BL/6J mice were intragastrically inoculated with Lmo-InlA-mur-lux and Lmo-EGD-lux and bacterial dissemination to internal organs was analysed using bioluminescent *in vivo* imaging (BLI). These mouse inbred strains were chosen for the study as they represent priority strains for the mouse phenome project [18] and their degree of host resistance to oral *L. monocytogenes* infection has never been investigated and compared in a single study under identical infection challenge conditions. We report here that infection with murinised *Listeria* resulted in earlier onset of listeriosis compared to infections with the non-murinised *Listeria* strain in different mouse genetic backgrounds. BLI enabled accurate measurement of bacterial dissemination over consecutive days in the acute stage of disease and showed that Lmo-InlA-mur-lux disseminated earlier from the intestine to target organs in the C3HeB/FeJ, A/J, and BALB/cJ mice. However, no increase in dissemination to the brain was detected, revealing that *Listeria* uses different mechanisms to cross the intestinal epithelium and to cross the blood–brain barrier.

## Results

### Dynamics of Lmo-InlA-mur-lux and Lmo-EGD-lux dissemination visualized by BLI

**Figure 1:**
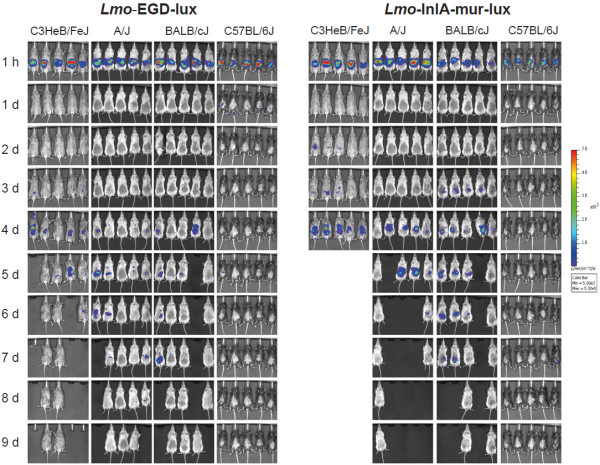
**Bioluminescence imaging (BLI) of listeriosis in different inbred mouse strains after oral infection challenge with Lmo-EGD-lux and Lmo-InlA-mur-lux.** Ten female C3HeB/FeJ, A/J OlaHsd, BALB/cJ and C57BL/6J mice were intragastrically challenged with 5 × 10^9^ CFU Lmo-EGD-lux (left column) or Lmo-InlA-mur-lux (right column) and the progress of infection was assessed by BLI for 9 days. Bacterial luciferase activity was visualized in five mice per measurement using the IVIS 200 imaging system as described in Methods. Serial BLI data are shown for a set of five mice for a time period of 9 days p.i.. They are representative of two independent experiments each with a total of 10 mice per inbred mouse strain. Empty spaces indicate dead mice. The colour bar indicates photon emission with 4 min integration time in photons/s/cm^2^/sr.

**Figure 2:**
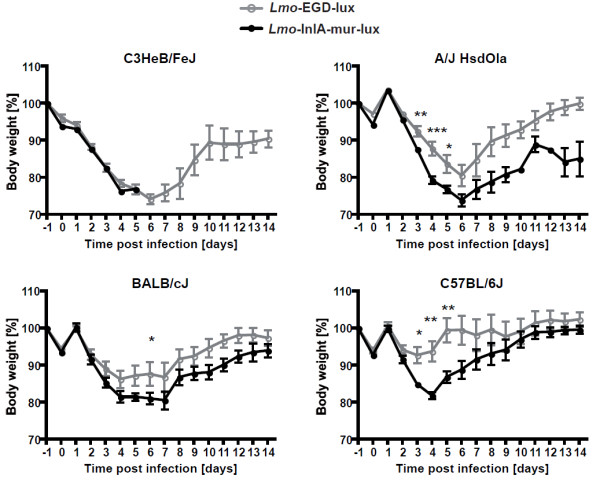
**Body weight changes of different mouse inbred strains after oral infection with 5 × 10**^**9**^ **CFU Lmo-EGD-lux and Lmo-InlA-mur-lux.** Ten female C3HeB/FeJ, A/J OlaHsd, BALB/cJ, and C57BL/6J mice were intragastrically infected with 5 × 10^9^ CFU Lmo-EGD-lux (grey graphs) or Lmo-InlA-mur-lux (black graphs). Body weight changes were monitored daily over 14 days. The weight loss on the day of infection, day 0, is due to overnight starving of the mice. After intragastric infection challenge mice had again access to food ad libitum. Data are representative of two independent experiments with groups of 10 mice per inbred mouse strain. Data represent means ± SEM, *p < 0.05; **p < 0.01; ***p < 0.001.

To compare the dissemination dynamics of the murinised and wildtype *L. monocytogenes* strains in different inbred genetic backgrounds, C57BL/6J, C3HeB/FeJ, A/J, and BALB/cJ female mice (n = 10) were intragastrically infected with 5 × 10^9^ CFU of either Lmo-InlA-mur-lux or Lmo-EGD-lux. BLI was first performed 1 h post infection, and then daily over a period of 9 days using identical IVIS settings for every mouse. As an additional parameter for the course of infection body weight was recorded daily. Strong bioluminescence signals were detected in the abdomen 1 h after inoculation in all infected animals representing the inoculum (Figure 1). As reported previously [19], these light signals diminished to undetectable levels over the next 24 h. This reduction in light emission is largely caused by the passage of the bacteria from the stomach to the intestine and the overnight clearance of most of the bacteria by faecal shedding. Depending on the genetic background of the host and the listerial strain used in infections, the bioluminescent signals reappeared after 2 to 4 days p.i (Figure 1). This second reappearance of light signals took place earliest in a subset of the Lmo-InlA-mur-lux infected C3HeB/FeJ mice at 2 d.p.i. becoming stronger during the next 24 h of infection until clearly detectable in all infected C3HeB/FeJ mice (Figure 1). At 4 d.p.i. bioluminescent signals were detected in the intestine, mesenteric lymph nodes (MLN), liver, and gallbladder of Lmo-InlA-mur-lux infected C3HeB/FeJ mice indicating that at this timepoint murinised *Listeria* had disseminated systemically from the intestine to the deep organs (Figure 1). This dissemination accompanied rapid onset of listeriosis symptoms in Lmo-InlA-mur-lux infected C3HeB/FeJ with reduced behavioural activity and dramatic losses in body weight (Figure 2). In contrast, in Lmo-EGD-lux infected C3HeB/FeJ mice BLI signals reappeared one day later at 3 d.p.i. in a subset of animals (Figure 1). Signals were first detectable in the small intestine, MLNs and gallbladder, then at 4 and 5 days p.i. also in the liver. Lower intensities were observed compared to signals measured in Lmo-InlA-mur-lux infected C3HeB/FeJ mice (Figure 1, and Additional file 1: Figure S1) and correlated with a delayed onset of listeriosis symptoms. Similar trends were seen in A/J and BALB/cJ mice with mice infected with the murinised strain showing bioluminescence earlier and in a wider range of organs (Figure 1). The more increased bioluminescence signal in Lmo-InlA-mur-lux infected A/J and BALB/cJ mice compared to Lmo-EGD-lux infected animals was paralleled in body weight changes (Figure 2). In C57BL/6J infected mice bioluminescent signals were first detectable in Lmo-EGD-lux and Lmo-InlA-mur-lux infected cohorts in the abdomen at 1 d.p.i. (Figure 1). These light signals were not further detectable at 2 d.p.i., however in a small subset of Lmo-EGD-lux and Lmo-InlA-mur-lux infected C57BL/6J mice small areas of light emission were detectable on days 4, 5, 6 and 8 post infection (Figure 1). *Ex vivo* imaging of dissected organs suggested that these light signals were emitted from the gallbladder (Additional file 2: Figure S2). As the sensitivity of the IVIS 200 was set to a fixed value for all animals imaged regardless of the mouse strain investigated, light signals were more quenched in pigmented C57BL/6J mice as compared to the other inbred strains. Among all investigated mouse inbred strains, C57BL/6J mice were found to be most resistant to infection with Lmo-EGD-lux and Lmo-InlA-mur-lux which was reflected in increased survival rates and better post infection recovery (Figure 2 and Additional file 3: Figure S3).

In summary, the whole animal BLI of Lmo-InlA-mur-lux and Lmo-EGD-lux infected C57BL/6J, C3HeB/FeJ, A/J, and BALB/cJ mice showed that infection with ‘murinised’ *Listeria* were associated with stronger and earlier bioluminescent signals compared to infections with the ‘non-murinised’ *L. monocytogenes* strain and enabled accurate and repeated tracking of bacterial dissemination. C57BL/6J mice were most resistant to orally acquired listeriosis whereas C3HeB/FeJ mice were most susceptible.

### Quantification of Lmo-InlA-mur-lux and Lmo-EGD-lux tissue burden after oral infection in different inbred mouse strains

**Figure 3:**
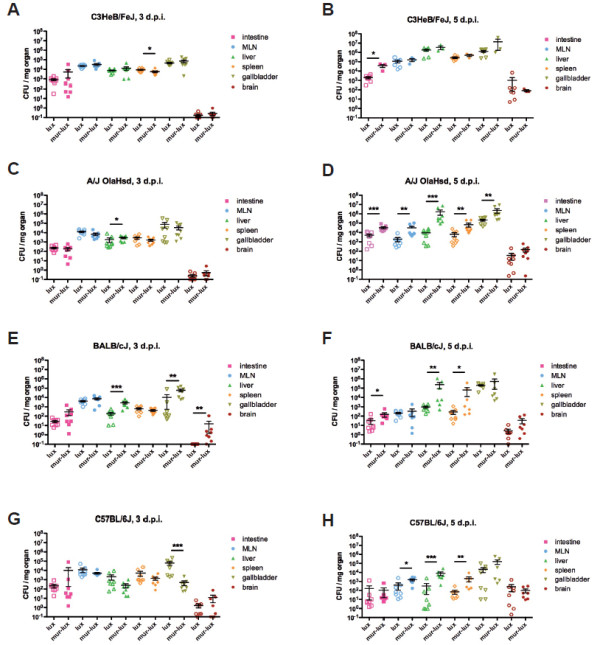
**Kinetics of bacterial organ colonization in different inbred mouse strains after intragastric infection challenge with Lmo-EGD-lux and Lmo-InlA-mur-lux.** Female C3HeB/FeJ (**A,B**), A/J OlaHsd (**C,D**), BALB/cJ (**E,F**) and C57BL/6J (**G,H**) were intragastrically challenged with 5 × 10^9^ CFU Lmo-EGD-lux (open symbols) or Lmo-InlA-mur-lux (filled symbols). At indicated times post infection a group of 8 mice were sacrificed and organs (small intestine, mesenteric lymph nodes = MLN, liver, spleen, gallbladder and brain) were prepared, homogenized and plated on BHI agar plates and CFU/mg organ was determined. Mean CFU (horizontal lines) with standard error of the mean are shown on day 3 (left column) and day 5 (right column) post infection. Note, on day 5 p.i. most of the C3HeB/FeJ mice had already been euthanized due to development of severe listeriosis. Significant differences between Lmo-EGD-lux and Lmo-InlA-mur-lux bacterial tissue loads are indicated as *p < 0.05; **p < 0.01, and ***p < 0.001 (data represent means ± SEM, non-parametric Mann–Whitney-*U*-test). Data are representative of two independent experiments.

We determined the bacterial loads in different *L. monocytogenes* target organs at 3 and 5 days p.i. as the onset of clinical symptoms of listeriosis and body weight changes indicated these timepoints were most critical for the course of infection. Again, females of the C3HeB/FeJ, A/J, BALB/cJ and C57BL/6J strains were intragastrically inoculated with either 5 × 10^9^ CFU of Lmo-InlA-mur-lux or Lmo-EGD-lux and organ loads analysed by plating tissue homogenates on BHI agar plates (Figure 3). In C3HeB/FeJ mice, high organ loads of 10^3^-10^4^ CFU for Lmo-InlA-mur-lux and Lmo-EGD-lux were measured at 3 d.p.i. in the small intestine, liver and spleen and most particularly for both bacterial strains in the gallbladder and MLNs (10^4^-10^5^ CFU). In contrast, no substantial CFUs were detectable in C3HeB/FeJ brains for either bacterial strain at this timepoint. At 5 d.p.i., bacterial loads in C3HeB/FeJ mice reached 10^5^-10^7^ CFU in MLNs, liver, gallbladder, and spleen showing that both listerial strains were replicating at high levels in most internal organs. In A/J mice significantly higher Lmo-InlA-mur-lux loads were measured at 3 d.p.i. in the liver as compared to Lmo-EGD-lux loads (Figure 3). Bacterial loads of Lmo-InlA-mur-lux in A/J mice increased tenfold from 3 to 5 days p.i. in the gallbladder, small intestine, and spleen, and 100-fold in the liver and brain. Consistently higher CFU counts were measured in Lmo-InlA-mur-lux infected A/J mice as compared to Lmo-EGD-lux infected animals in most internal organs. However, no differences in brain CFU loads were detectable in A/J mice infected with Lmo-EGD-lux or Lmo-InlA-mur-lux at this timepoint (Figure 3).

Similarly, in resistant C57BL/6J mice bacterial loads between Lmo-InlA-mur-lux and Lmo-EGD-lux infected mice were not significantly different at 3 d.p.i., with exception of the gallbladder. However, at 5 d.p.i., higher Lmo-InlA-mur-lux CFU counts were found in MLNs, liver and spleen as compared to Lmo-EGD-lux organ loads. In comparison to the susceptible C3HeB/FeJ and A/J strains, bacterial loads in internal organs of C57BL/6J mice were in general 10-100 fold lower for both listerial strains. Thus, in comparison to the other investigated inbred strains, C57BL/6J mice have an enhanced capability to control *L. monocytogenes* dissemination and replication in target organs but still show the increased susceptibility to the murinised strain. BALB/cJ mice displayed an intermediate resistance to *Listeria*. Significant differences in bacterial burden between Lmo-InlA-mur-lux and Lmo-EGD-lux infected BALB/cJ mice were detected at 3 d.p.i. in the liver, gallbladder, and brain. At 5 d.p.i., Lmo-InlA-mur-lux bacterial loads remained higher in the small intestine, liver, and spleen compared to Lmo-EGD-lux loads, however, no further CFU differences were detected in the brain for both *L. monocytogenes* strains.

Taken together, the analysis of bacterial replication kinetics in different internal organs demonstrated, in general, higher levels of Lmo-InlA-mur-lux bacterial loads compared to Lmo-EGD-lux loads across the different mouse inbred strains analysed. Host resistance of C57BL/6J mice against *Listeria* correlated with the ability to control *L. monocytogenes* replication in target organs whereas in susceptible C3HeB/FeJ, A/J, and BALB/cJ mice *Listeria* replication was less efficiently controlled. From all mouse inbred strains investigated, C3HeB/FeJ mice displayed the highest bacterial tissue burden and were thus found to be most susceptible to Lmo-InlA-mur-lux and Lmo-EGD-lux infection.

### Histopathological analysis of liver and spleen in Lmo-InlA-mur-lux and Lmo-EGD-lux infected C3HeB/FeJ and C57BL/6J mice

We analysed histopathological changes in liver and spleen of Lmo-InlA-mur-lux and Lmo-EGD-lux infected C3HeB/FeJ and C57BL/6J mice at 3 and 5 days p.i. We focused this comparative analysis on C3HeB/FeJ and C57BL/6J mice since they represent the two extremes of host susceptibility and resistance, respectively.

**Figure 4:**
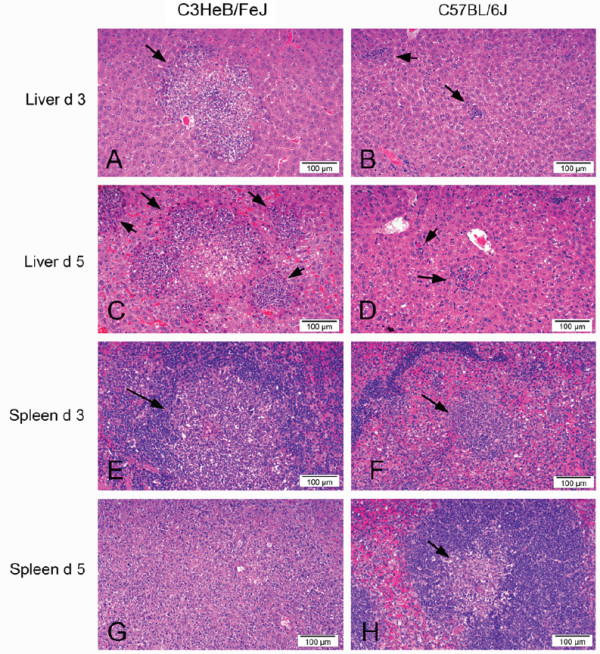
**Susceptibility of C3HeB/FeJ mice to orally acquired listeriosis correlates with severe necrotic lesions in liver and spleen.** Photographs of haematoxylin and eosin stained sections of liver (**A** to **D**) and spleen (**E** to **H**) from C3HeB/FeJ mice and C57BL/6J mice at three and five days post oral infection with *L. monocytogenes*. There are multifocal to coalescing areas of hepatic and splenic necrosis accompanied by neutrophils, macrophages and lymphocytes (arrows). The lesions are substantially more extensive in C3HeB/FeJ mice, and increase in severity from day 3 to day 5 p.i. In **4G** the splenic necrosis in the C3HeB/FeJ mice has expanded to entirely efface the normal splenic architecture, while in the C57BL/6J mice (**4H**) the lesion has progressed to a focal aggregate of macrophages with minimal necrosis. The images presented are representative of changes seen in both Lmo-InlA-mur-lux and Lmo-EGD-lux infected animals (**A**: EGD-lux; **B**: InlA-mur-lux; **C**: EGD-lux; **D**: EGD-lux; **E**: EGD-lux; **F**: InlA-mur-lux, **G**: EGD-lux; **H**: InlA-mur-lux).

The histopathological changes mirrored those seen in the BLI imaging with more numerous and severe lesions present in the liver and spleen of C3HeB/FeJ mice compared to C57BL/6J mice. However, there was no detectable difference in the pathology identified in mice inoculated with Lmo-InlA-mur-lux or Lmo-EGD-lux. The changes in the liver of the C57BL/6J mice at day 3 and 5 p.i. consisted of randomly scattered, small, focal aggregates of macrophages, neutrophils and occasional lymphocytes accompanying a small number of necrotic hepatocytes (Figure 4B and D). The pathological changes in the livers of C3HeB/FeJ mice were substantially more numerous and extensive at both days 3 and 5 p.i., characterised by randomly scattered areas of necrosis up to 200 μm in diameter, cuffed by numerous neutrophils (often degenerate), macrophages and lymphocytes (Figure 4A and B). In the spleen the lesions were again more numerous and severe in the C3HeB/FeJ mice compared to the C57BL/6J mice at both days 3 and 5 post infection. At 3 d.p.i. the spleens from C3HeB/FeJ mice contained more numerous and larger areas of necrosis, mainly affecting the white pulp areas of the spleen, accompanied by cellular debris, neutrophils and macrophages (Figure 4E and F). By 5 d.p.i. there was minimal necrosis remaining in the resolving lesions in the spleens of C57BL/6J mice (Figure 4H), however the pathology in the C3HeB/FeJ mice had increased in severity to efface entire lymphatic nodules (Figure 4G).

### Increased susceptibility of C3HeB/FeJ mice to oral *Listeria* challenge correlates with elevated inflammatory responses

**Figure 5:**
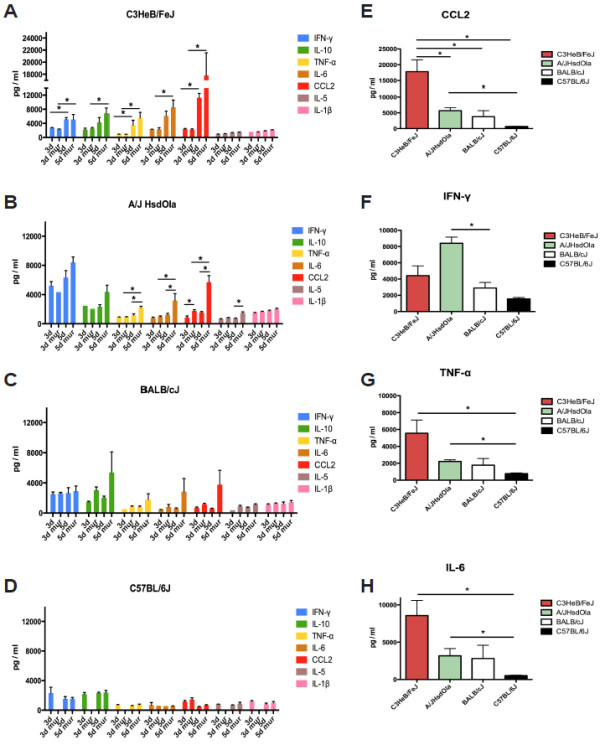
**Chemokine and cytokine production of different mouse inbred strains after oral infection with Lmo-EGD-lux and Lmo-InlA-mur-lux.** Female C3HeB/FeJ (**A**), A/J OlaHsd (**B**), BALB/cJ (**C**) and C57BL/6J mice (**D**) were orally infected with 5 × 10^9^ CFU Lmo-EGD-lux or Lmo-InlA-mur-lux. Blood samples were collected at 3 and 5 d.p.i. and cytokine and chemokine levels were determined using Luminex bead assays. 3d and 5d indicate Lmo-EGD-lux infected animals at 3 and 5 d.p.i., respectively; 3d mur and 5d mur indicate Lmo-InlA-mur-lux infected animals at these timepoints (n = 8). For each timepoint, chemokine and cytokine concentrations were determined in triplicate for each inbred mouse and *L. monocytogenes* strain. Data represent means ± SEM. (**E-H**) Comparison of chemokine and cytokine production across Lmo-InlA-mur-lux infected mice from the different inbred mouse strains at 5 d.p.i.. Shown are statistical significant differences of indicated cytokine and chemokine levels in the peripheral blood between groups of mice of the analysed inbred mouse strains. Data represent means ± SEM; *p < 0.05, non-parametric Mann–Whitney-*U*-test. One out of two representative experiments is shown (**A-H**).

To investigate differential inflammatory responses associated with Lmo-InlA-mur-lux and Lmo-EGD-lux infections, we measured serum levels of IFN-γ, IL-10, TNF-α, IL-6, CCL2, IL-5 and IL-1β at 3 and 5 days p.i. using Luminex bead arrays (Figure 5). Differences in the level of pro-inflammatory cytokines and chemokines between Lmo-InlA-mur-lux and Lmo-EGD-lux infected animals were not apparent at 3 d.p.i. but became detectable at 5 days post infection. A/J showed the largest difference in the level of TNF-α, IL-6, and CCL2 production between Lmo-InlA-mur-lux and Lmo-EGD-lux inoculated animals. A more subtle difference in the level of these three cytokines was also apparent in C3HeB/FeJ and BALB/cJ mice. IL-5 and IL-1β levels did not change during the course of infection across the different inbred strains (Figure 5A-D), however, CCL2 levels increased dramatically in Lmo-InlA-mur-lux infected C3HeB/FeJ mice from day 3 to 5 p.i. and to a lesser extent also in Lmo-InlA-mur-lux infected A/J and BALB/cJ over this time period (Figure 5A-D). In contrast, resistant C57BL/6J mice displayed low serum levels of IFN-γ, TNF-α, IL-6, and CCL2 at both timepoints of infection. There was also no increase in the level of these cytokines and CCL2 from day 3 to 5 p.i. in either Lmo-InlA-mur-lux or Lmo-EGD-lux infected C57BL/6J mice demonstrating the tight control of inflammatory responses in this mouse inbred strain. The differences in production of these cytokines and CCL2 in the different inbred mouse strains were most apparent in Lmo-InlA-mur-lux infected animals at 5 d.p.i. Compared to all other mouse strains, susceptible C3HeB/FeJ mice displayed the highest levels of CCL2, TNF-α, and IL-6 in the serum in response to Lmo-InlA-mur-lux infection (Figure 5E-H). Lmo-InlA-mur-lux infected A/J mice displayed high IFN-γ levels (Figure 5F) whereas C57BL/6J mice showed low serum concentrations for all of these cytokines and the CCL2 chemokine (Figure 5E-H). Thus, the elevated susceptibility of C3HeB/FeJ mice and their inability to control *Listeria* replication correlated with an exaggerated production of pro-inflammatory mediators. Serum levels of IL-10 were also high in Lmo-InlA-mur-lux infected C3HeB/FeJ mice (data not shown). However, this apparently did not result in downregulation of pro-inflammatory responses.

### Oral infection with murinised Lmo-InlA-mur-lux is associated with increased induction of interferon-β

An important factor which determines the virulence of *Listeria monocytogenes* is the amount of type I interferons produced in the host during infection. High levels of interferon-β (IFN-β) have been demonstrated to be associated with host susceptibility to *Listeria* infection and mice deficient for IFN-β signalling components such as the *type I interferon receptor* (*Ifnar*) gene or the *interferon regulatory factor 3* (*Irf3*) gene are more resistant to lethal *L. monocytogenes* infection [20-25]. Furthermore, variations in the induction of IFN-β responses in the host by different *Listeria* strains have been linked with differences in strain virulence [26-29]. To analyse and compare kinetics of *Ifnb1* induction after intragastric infection challenge with Lmo-InlA-mur-lux and Lmo-EGD-lux we developed a dual luciferase detection model. IFN-β-reporter mice were inoculated with 5 × 10^9^ CFU bacteria of each listerial strain and monitored both bacterial and firefly luciferase activity by BLI over a time course of 8 days. Bacterial and firefly luciferases have different peak emission length of 410 nm and 610 nm, respectively [30]. Importantly compared to the bacterial luciferase, the firefly luciferase has stronger light emitting activity and can be separately measured *in vivo* by BLI after systemic administration of its substrate luciferin. BLI signals from the bacterial luciferase can then be subtracted from firefly BLI signals for solely quantification of *Ifnb1* induction levels.

**Figure 6:**
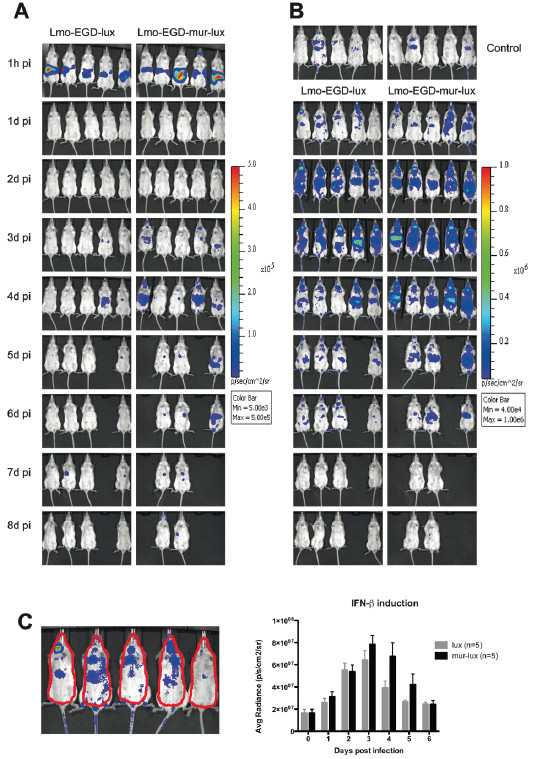
**Oral infection challenge with murinised Lmo-InlA-mur-lux is associated with elevated IFN-β induction.** Albino IFN-β-reporter (*Ifnb1*^*tm2.2Lien*^) mice on a C57BL/6J genetic background were infected intragastrically with 5 × 10^9^ CFU Lmo-EGD-lux or Lmo-InlA-mur-lux (n = 5). At the indicated timepoints, mice were first analysed for dissemination of bioluminescent *L. monocytogenes* as described for Figure 1 (**A**) and then subsequently i.v. injected with luciferin and monitored for firefly luciferase activity as a reporter of IFN-β induction (**B**), see Methods. Serial BLI for bacterial and firefly luciferase activity was performed at 1 h post infection and then daily till 8 d.p.i.. The colour bar indicates photon emission with 4 min integration time in photons/s/cm^2^/sr. Uninfected *Ifnb1*^*tm2.2Lien*^ reporter mice are shown as controls at the top in (**B**). (**C**) Quantification of firefly luciferase light signals presented in (**B**) in Lmo-EGDe-lux (grey columns) and Lmo-InlA-mur-lux (black columns) infected IFN-β-reporter mice by measuring luminescence intensity in an identical selected region in each animal as indicated on the left. Data represent means ± SEM. Bacterial luciferase photon emission was subtracted from firefly BLI signals to generate the graph shown in (**C**). One out of two representative experiments is shown (**A**-**C**).

One day after inoculation with Lmo-InlA-mur-lux or Lmo-EGD-lux we detected the first firefly luciferase signals in the spleen and cervical lymph nodes (Figure 6B) as described previously for an intravenous *Listeria* infection model [24]. At this timepoint, light signals from replicating bacteria were not yet visible (Figure 6A). Host bioluminescent signals had similar intensities in Lmo-InlA-mur-lux and Lmo-EGD-lux infected IFN-β-reporter mice at 24 h p.i., although two out of five Lmo-InlA-mur-lux infected animals showed a more intensive induction of the IFN-β-reporter compared to Lmo-EGD-lux infected animals (Figure 6B). At 2 d.p.i., IFN-β reporter signals in mice infected with either bacterial strain were further increased and then also detectable in the intestine and the liver. The intensities of the firefly luciferase signals increased further at days 3 and 4 p.i. and became more pronounced in Lmo-InlA-mur-lux infected mice as compared to Lmo-EGD-lux infected animals (Figure 6B and C). At 5 d.p.i., two Lmo-InlA-mur-lux and one Lmo-EGD-lux infected mice which had displayed high IFN-β reporter signals on earlier timepoints of the infection developed severe listeriosis (Figure 6B) and succumbed to the infection or had to be euthanized for ethical reasons. This demonstrated, in line with previous studies, that high levels of IFN-β production are associated with elevated mortality rates in listeriosis [31,32]. Overall, in the Lmo-InlA-mur-lux infected experimental cohort, 3 out of 5 mice succumbed to the infection whereas in the Lmo-EGD-lux experimental cohort only 1 animal out of 5 did not survive the infection. This demonstrated, in line with previous studies, that high levels of IFN-β production are associated with elevated mortality rates in listeriosis [31,32]. Thus, taken together murinised *Listeria* induces higher levels of IFN-β in orally challenged mice compared to non-murinised *Listeria*.

### Oral infection challenge with ‘murinised’ *Listeria* does not result in increased neuroinvasion into the brain

*L. monocytogenes* can induce meningitis, meningoencephalitis, and rhombenencephalitis in infected humans and animals [33]. It is currently not well understood which virulence factors of *L. monocytogenes* control the invasion of the pathogen into the central nervous system (CNS). InlA- and InlB-dependent uptake mechanisms have been suggested for direct invasion of *L. monocytogenes* into brain microvascular endothelial cells and choroid plexus epithelial cells [34,35]. Our murinised *Listeria* infection model is permissive for InlA- and InlB-mediated invasion mechanisms and allows investigation of the role of InlA-Cdh1 interactions in listerial brain tropism. To test the hypothesis that InlA-Cdh1 interactions contribute to the invasion of *L. monocytogenes* into the brain we paid particular attention to the development of neurological abnormalities in Lmo-InlA-mur-lux and Lmo-EGD-lux infected mice. Interestingly, mice displaying abnormal neurological behaviour such as circling, head tilting or ataxia were very rarely observed. From a total of 290 mice that were orally challenged with Lmo-InlA-mur-lux and Lmo-EGD-lux (5 × 10^9^ CFU) and monitored for clinical symptoms only 3 animals developed neurological phenotypes (Table 1). These affected mice were identified in the A/J, BALB/cJ, and C57BL/6J inbred strains and occurred with equally low frequency in both Lmo-InlA-mur-lux and Lmo-EGD-lux challenged animals (Table 1). In these cases the appearance of neurological symptoms occurred at 7 d.p.i.. As described above, no major differences in bacterial brain loads were observed between Lmo-InlA-mur-lux and Lmo-EGD-lux challenged mice across the different investigated inbred strains (Figure 3). This was also true for the 7 d.p.i. timepoint when we did observe the above described rare neurological phenotypes in single mice of the C57BL/6J, A/J and BALB/cJ inbred strains but no differences in brain CFU loads among all cohorts of Lmo-InlA-mur-lux and Lmo-EGD-lux infected mice were detected (data not shown). Although, Lmo-InlA-mur-lux and Lmo-EGD-lux infected BALB/cJ mice did show significant differences in brain CFUs at 3 d.p.i. with higher loads of murinised Lmo-InlA-mur-lux bacteria, these differences were not further detectable at 5 and 7 days p.i.. We therefore conclude that at least in our infection system, InlA-Cdh1 interactions do not play a role in the dissemination of *L. monocytogenes* to the brain. Moreover, even in different mouse genetic backgrounds no evidence for InlA-mediated CNS infection was found.

**Table 1:** Neurological symptoms in mouse inbred strains after oral infection with Lmo-InlA-mur-lux and Lmo-EGD-lux

***L. monocytogenes *strain**	**Mouse inbred strain**	**Total number of mice infected**	**Number of mice displaying neurological abnormalities**	**Symptoms occurrence time post infection [days]**
*Lmo*-InlA-mur-lux	C3HeB/FeJ	40	0	
	A/J OlaHsd	30	1	7
	BALB/cJ	30	0	
	C57BL/6J	30	1	7
		**∑ 130**	**∑ 2**	
*Lmo-*EGD-lux	C3HeB/FeJ	40	0	
	A/J OlaHsd	40	0	
	BALB/cJ	40	1	7
	C57BL/6J	40	0	
		**∑ 160**	**∑ 1**	

## Discussion

*In vivo* bioluminescence imaging is an important technology for the spatial-temporal monitoring of infection processes that underlie microbial pathogenesis and host defence mechanisms [30,36]. Importantly, BLI allows repeated non-invasive imaging of pathogen dissemination to target organs and was used to identify the murine gallbladder as a novel organ of infection and as a host reservoir for extracellular *Listeria* replication and pathogen shedding [19,37]. In the present study, we have combined an InlA and InlB permissive mouse infection model of *L. monocytogenes*[12] with BLI, bacterial growth, and histopathology. We accurately compared resistance of mouse strains of different genetic backgrounds to orally acquired murinised and non-murinised *Listeria*. We identified the C3HeB/FeJ, A/J, and BALB/cJ strains as being susceptible to oral *L. monocytogenes* challenge whereas C57BL/6J mice were resistant. BLI analysis was more sensitive than bacterial culture or histopathology at detecting differences in pathogenesis between the murinised and non-murinised *Listeria* strains, and demonstrated that in the susceptible mouse inbred strains Lmo-InlA-mur-lux spread to internal organs more quickly and in higher numbers when compared to Lmo-EGD-lux infected animals. Thus, murinised *Listeria* can efficiently be used with mice of different genetic backgrounds for studies on mechanisms of orally acquired listeriosis. Importantly, once the intestinal barrier has been overcome by the pathogen, patterns of *L. monocytogenes* host resistance that have been previously determined by using systemic infection models are very similar to those that were observed in our present study. C57BL/6J mice are resistant to both oral and intravenous *L. monocytogenes* infection challenge, whereas C3HeB/FeJ, A/J and BALB/cJ mice are highly susceptible in both mouse infection models [38-42]. We show here that the host resistance of C57BL/6J mice to intragastric *L. monocytogenes* infection correlates with moderate losses in body weight, increased survival, lower bacterial loads in internal target organs, and low levels of IL-6, TNF-α, IFN-γ, and CCL2 production as compared to the other inbred mouse strains investigated. The wide spectrum of *L. monocytogenes* host resistance in different inbred mouse strains is controlled by multiple genetic loci and complex interactions of different alleles impact on the overall phenotype of resistance or susceptibility towards *Listeria*[43]. Importantly, the differences in host resistance to oral Lmo-InlA-mur-lux infection, that have been investigated in this study across the four inbred strains, are unlikely to be causatively linked to polymorphisms in the *E-cadherin* gene. Although A/J and BALB/cJ mice carry private missense polymorphisms in *Cdh1*, the underlying coding changes (R6H, P267A, P267Q, F272S, A636G) are unlikely to impact on the function of the protein. Provean predictions indicated that these changes would be well tolerated and would not alter the function of the protein [44].

Classic genetic studies carried out almost 30 years ago by using recombinant inbred mouse strains identified the *Hc* locus as a contributor to listeriosis susceptibility in A/J mice. The *Hc* locus encodes the C5 complement protein and A/J mice have a C5 deficiency due to a two base pair intragenic deletion in the *Hc hemolytic complement* gene [41,45,46]. Consequently, A/J mice are relatively inefficient at recruiting inflammatory effector cells such as neutrophils and macrophages to the site of infection [47,48]. Differential host resistance to *L. monocytogenes* infection in BALB/cByJ and C57BL/6ByJ mice has been found to be genetically controlled by the *Listr1* and *Listr2* quantitative trait loci (QTLs) on mouse chromosomes 5 and 13, respectively [38]. Although, the underlying genes and molecular mechanisms of these QTLs in controlling *L. monocytogenes* host resistances have not been unravelled yet, it has been demonstrated recently that a polymorphism in intron 5 of the *interferon regulatory factor 3* gene (*Irf3*) on mouse chromosome 7 in the ByJ substrain of the C57BL/6 inbred strains contributes to *Listeria* host resistance of C57BL/6ByJ mice [22].

We have identified C3HeB/FeJ mice to be extremely susceptible to oral *L. monocytogenes* infection. C3HeB/FeJ were found to be sensitive to Lmo-InlA-mur-lux and Lmo-EGDx-lux infections, although Lmo-InlA-mur-lux showed also enhanced virulence in this mouse strain. The increased host susceptibility of C3HeB/FeJ mice correlated with high bacterial burdens in the small intestine, MLNs and deep organs and was associated with massively elevated inflammatory responses when compared to the other investigated inbred mouse strains. C3HeB/FeJ mice developed necrotic lesions in the spleen and liver in the early phase of the infection, and the size and number of these lesions correlated with listeriosis severity and mortality. Compared to the other inbred mouse strains investigated, C3HeB/FeJ mice displayed elevated serum levels of IL-6, TNF-α and extremely high levels of the chemokine CCL2 at 3 and 5 days post infection. CCL2 has been demonstrated to have an important role in defence against *L. monocytogenes* infection. It is highly upregulated during the early phase of *L. monocytogenes* infection and attracts inflammatory monocytes, T lymphocytes, and natural killer cells to the site of microbial infection [49-51]. In the spleen, CCL2 is produced by ERTR-9+ marginal zone macrophages which are early targets of *L. monocytogenes* infection and are crucial for innate immune defence [52]. High levels of CCL2, as for example induced by over expression in transgenic mice, have been demonstrated to be associated with increased sensitivity to *L. monocytogenes* infection [53]. Thus, elevated CCL2 levels in C3HeB/FeJ mice are likely to contribute to the overall increased detrimental inflammatory response that we have observed in these mice. However, this cannot explain the general host susceptibility of this mouse strain. Importantly, C3HeB/FeJ mice are susceptible to many pathogens including *Mycobacterium tuberculosis*[54], *Salmonella* Typhimurium [55,56], *Plasmodium chabaudi*[57], *Trypanosoma rhodesiense*[58], *Listeria monocytogenes*[59], and *Streptococcus pyogenes*[60,61]. Susceptibility to *M. tuberculosis* and *L. monocytogenes* infection in C3HeB/FeJ mice correlates with induction of severe necrotic lesions in the lung or liver and spleen, respectively [54,59]. The multifocal abscess formation in both mouse infection models is controlled by the *sst1* (*supersusceptibility to tuberculosis*) locus on mouse chromosome 1. *Sst1* encodes the Sp110/Ipr1 nuclear body protein which belongs to the SP100/SP140 family of nuclear body proteins [54,62]. The type I and II interferon inducible *Sp110/Ipr1* gene is not expressed in C3HeB/FeJ mice due to a complex structural rearrangement at the *Sst1* locus which left incomplete copies of the *Sp110/Ipr1* gene in this mouse strain [54,62]. Consequently, mice which carry the *Sst1* susceptibility allele are impaired in their innate immune response against intracellular pathogens such as *M. tuberculosis* and *L. monocytogenes*.

Another host factor which greatly influences susceptibility to *L. monocytogenes* infection is the amount of interferon-β produced in response to infection [20,21,23,28,31,32]. Production of interferon-β induces further release of type I interferons via autocrine and paracrine loops which can be detrimental due to induction of apoptosis in T cells and macrophages [63]. In addition, interferon-β is a major driver of TNF-α induced lethal shock by enhancing apoptosis of enterocytes and hepatocytes which results in bowel and liver damage [31]. We have compared induction of interferon-β responses in Lmo-InlA-mur-lux and Lmo-EGD-lux infected mice by using a luciferase reporter system and BLI *in vivo* imaging. Although we used *Infb1*-reporter mice on the *L. monocytogenes* resistant C57BL/6J genetic background we detected stronger signals of *Infb* induction in Lmo-InlA-mur-lux infected reporter mice as compared to Lmo-EGD-lux infected animals at days 4 and 5 post infection. The induction of *Infb1* correlated with the systemic dissemination of Lmo-InlA-mur-lux bacteria from the intestine to internal organs as these were detected earlier by BLI analysis of the bacterial luciferase reporter gene in Lmo-InlA-mur-lux infected animals compared to Lmo-EGD-lux infected mice.

In the present study we were not able to detect differences in the Lmo-InlA-mur-lux and Lmo-EGD-lux invasion of the brain amongst the different mouse inbred strains investigated. Invasion of the CNS after oral infection with *L. monocytogenes* is still poorly understood despite the importance of neurological complications in fatal cases of listeriosis [33]. Different hypotheses for routes of listerial neuroinvasion have been suggested including a retrograde transport of the *L. monocytogenes* from the oral epithelium to the rhombencephalon in cranial nerves [64,65] or dissemination of bacteria by the hematogenous route across the blood–brain barrier (BBB), either directly by extracellular bacteria in the blood [66] or via a Trojan horse mechanism by which intracellular bacteria are transported by infected leukocytes across the BBB [67-69]. Within the BBB, cells of the microvascular endothelium and the choroid plexus epithelium express both host receptors, Cdh1 and Met, for InlA and InlB, respectively [33]. Thus, theoretically these cells should be accessible for InlA- and InlB-mediated *L. monocytogenes* invasion. However, in our study we did not find any evidence for an InlA-dependent brain invasion mechanism. The occurrence of neurolisteriosis as indicated by abnormal neurological behaviour of mice after oral infection with Lmo-InlA-mur-lux or Lmo-EGD-lux was an extremely rare event. From a total of 290 analysed animals, only three mice displayed ataxia or circling behaviour after infection. Two of these animals had been infected with Lmo-InlA-mur-lux and one mouse with Lmo-EGD-lux. All three affected animals were from different inbred mouse strains. Furthermore, our analysis of Lmo-InlA-mur-lux and Lmo-EGD-lux bacterial loads in the brain did not detect major differences between both listerial strains. Although, BALB/cJ mice did show higher bacterial loads for Lmo-InlA-mur-lux at 3 d.p.i. in the brain, they were not longer detectable by 5 and 7 d.p.i., and had no effect on the prevalence of neurological symptoms in this mouse strain. Therefore, we conclude that at least in our murinised *L. monocytogenes* infection model, InlA-Cdh1 interactions do not play a role in *Listeria* CNS neuroinvasion.

By using a new, natural *L. monocytogenes* infection model which involved feeding of contaminated food to mice, Bou Ghanem and colleagues have very recently shown that InlA is not required for the initial establishment of intestinal infection in mice [70]. However, expression of the murinised InlA^m^ was demonstrated to be associated with increased bacterial persistence in the lamina propria and enhanced dissemination to mesenteric lymph nodes [70]. This new finding suggests that InlA is important for overcoming a bottleneck in the gut that then leads to the systemic spread of the pathogen. The E-cadherin expressing host cells that are used by *Listeria* to overcome this bottleneck have not yet been identified. Although, preliminary finding suggest that these cells might be monocyte-derived migratory phagocyte that express E-cadherin. Future experiments incorporating conditional ablation of E-cadherin in different cells types (e.g. in enterocytes, macrophages, and dendritic cells) with murinised *L. monocytogenes* will help verify the existence of this hypothetical cell population.

## Conclusion

In summary, we conclude that the murinised, bioluminescent *L. monocytogenes* strain provides a versatile tool to analyse the pathogenesis of listeriosis in a range of different mouse model systems. By comparing the host resistance to orally acquired listeriosis in four inbred strains of mice we identified C57BL/6J mice to be most resistant to infections whereas BALB/cJ, A/J and C3HeB/FeJ were identified to be susceptible. Importantly, we did not find evidence in any of the investigated diverse mouse genetic backgrounds that expression of murinised InlA enhanced listerial invasion into the brain, revealing that *Listeria* uses a different invasion mechanism in different target organs. It is unlikely that InlA-Cdh1 interactions are a major driver of neurolisteriosis.

## Methods

### Mice

Female inbred A/J OlaHsd (Harlan-Winkelmann, Venray, Netherland), BALB/cJ, C57BL/6J (Janvier, St. Berthevin Cedex, France) and C3HeB/FeJ (Charles River, Sulzfeld, Germany) mice were obtained at 9-10 weeks of age. IFN-β-reporter mice (*Ifnb1*^*tm2.2Lien*^) on an albino C57BL/6 (B6(Cg)-*Tyr*^*c-2J*^/J) genetic background have been described previously [24,71]. Briefly, in this transgenic mouse the firefly luciferase reporter gene is under the control of the *Ifnb* promoter. This allows the detection of *Ifnb* induction *in vivo* with BLI after intravenous injection of D-luciferin (see below). All mice were housed under specific-pathogen-free conditions at the Helmholtz Centre for Infection Research (Braunschweig, Germany). Mice were acclimatised for 1 to 2 weeks in the facility before being used in infection challenge studies. All experiments were performed in accordance to German laws and animal welfare regulations after approval was granted from the Niedersächsisches Landesamt für Verbraucherschutz und Lebensmittelsicherheit (LAVES) as the local authority. The license number for this study was 33.11.42502-04-098/07.

### Bacterial strains and growth conditions

*Listeria monocytogenes* EGDe-lux (Lmo-EGD-lux) and *L. monocytogenes* EGDe-InlA-mur-lux (Lmo-InlA-mur-lux) have been described previously [17]*.* Both strains are isogenic and have been tagged with the constitutive bioluminescent *lux* marker pIMK2*lux*[44]. The *inlA* of Lmo-InlA-mur-lux was modified by site directed mutagenesis to express the murinised form of InlA with codon changes Ser192Asn and Tyr369Ser [17]. Listerial strains were grown in an overnight culture of Brain Heart Infusion (BHI) medium with shaking at 37°C. The next morning, bacterial cultures were diluted 1:10 and grown in BHI broth at 37°C until mid-log phase was reached. Bacteria were then harvested by centrifugation, washed several times and resuspended in sterile PBS. The numbers of colony forming units (CFU) of *L. monocytogenes* were determined by counting cells in a THOMA-chamber and by calculating the appropriate number of bacteria for infection. Plating bacteria on BHI agar plates verified the actual number of CFU in the inoculum.

### Animal infection

Age matched groups of female mice (10-12 weeks), were prepared for infection challenge by withheld of food for 12 h; drinking water was replaced by carbonate buffered water (2,6% NaHCO_3_). Bacteria were prepared as described [12]. Briefly, a total of 0.2 ml of the desired inoculum of either strain was mixed with 0.3 ml PBS containing 50 mg CaCO_3_[15]. A suspension of 5 × 10^9^ CFU was inoculated intragastrically into mice using a 21-gauge feeding needle attached to a 1 ml syringe. After infection mice were given access to food and water ad libitum. For CFU determination, small intestines, mesenteric lymph nodes, spleens, livers, gallbladders and brain of sacrificed mice were aseptically removed. To determine only intracellular bacterial load in small intestines, organs were washed with PBS and incubated in DMEM containing 100 μg/ml gentamicin for 2 h to kill extracellular bacteria. Serial dilutions of homogenates were plated on BHI agar plates and colonies were counted after overnight incubation at 37°C. All samples were weighted and homogenized in pre-cooled PBS.

For histopathological analysis of liver and spleen, organs were fixed in 10% buffered formalin, dehydrated, and embedded in paraffin. Sections of 4 μm were cut and stained with hematoxylin-eosin (H&E), and assessed blind by one researcher (PB) for evaluation of pathologic changes.

### *In vivo* imaging

For detection of bioluminescence, mice were anesthetized using isoflurane (Abbott Animal Health). Isoflurane gas anesthetic was administered at 2% in oxygen, which enables mild anaesthesia. BLI images were obtained using an IVIS 200 imaging system (CaliperLS) with integration time of 4 min at a binning of 8 and F/stop of 1. For the detection of *in vivo* enzymatic activity of the firefly luciferase, IFN-β-reporter mice were injected intravenously (i.v.) with 150 mg/kg of D-Luciferin (Synchem) in PBS, 5-10 min prior to imaging. Mice were anesthetized with isoflurane and monitored using the IVIS 200 imaging system according to manufactures instructions. Camera settings and exposure time were identical for all images. Photon flux was quantified by using the Living Image 3.1 software (CaliperLS).

### Luminex measurements of cytokines and chemokines

Cytokine and chemokine measurements were performed with a Luminex xMAP System using a Mouse Cytokine Twenty-Plex Antibody Bead Kit (Invitrogen). The following cytokines and chemokines were simultaneous quantified in single samples: IFN-γ, IL-10, TNF-α, IL-6, CCL2, IL-5 und IL-1β. Serum from indicated timepoints were collected and stored at -80°C. Cytokine and chemokine concentrations were determined in triplicates from at least 3 individuals of each mouse inbred strain. All procedures were carried out according to the manufacturer’s specifications (Invitrogen).

### Statistical analysis

Bacterial loads and cytokine/chemokine concentrations are depicted as mean +/- SEM. Statistical analysis of these data was performed using the Mann–Whitney U non-parametic test and the GraphPad Prism 5 (version 5.01) analysis software (GraphPad Software Inc.). Significance levels are depicted in figures as: *, P < 0.05; **, P < 0.01; ***, P < 0.001.

## Competing interests

The authors declare that they have no competing interests.

## Authors’ contributions

SB conducted all infection challenge experiments with help from BP. PMB performed the histopathological analysis. SB and SL conducted the BLI interferon-β reporter imaging and analysed the data. SW and CGMG contributed with mouse and *L. monocytogenes* strains and the reviewing of the manuscript. KS contributed to the study design, coordination of experiments and analysis of data. AL designed experiments, analysed data and drafted the manuscript. All authors read and approved the final manuscript.

## Supplementary Material

Additional file 1: Figure S1 Quantified BLI values from Figure 1. Light emission values from animals shown in Figure 1 were measured in an identical region in every mouse as shown in (A) and quantified as photons/s/cm^2^/sr. As described for Figure 1, mice from different inbred strains (n = 5, B-E) were intragastrically infected with 5 × 10^9^ CFU Lmo-EGD-lux (grey circles) or Lmo-InlA-mur-lux (black circles) and analysed for 9 days post infection. Click here for file

Additional file 2: Figure S2 *Ex vivo* BLI analysis of dissected internal organs. Six organs from Lmo-EGD-lux or Lmo-InlA-mur-lux infected animals (5 × 10^9^ CFU) were dissected at day 3 (3d) or day 5 (5d) post infection and imaged in an IVIS 200 imaging system. To aid interpretation of the figure a colour coded circle has been placed around each organ which emitted detectable light as shown in the example in (**A**). (**B**) Comparison of organ light emission signals in C3HeB/FeJ, A/J OlaHsd, BALB/cJ, and C57BL/6J female mice (n = 8, at day 0 of infection). The same imaging conditions were used for every organ by setting the IVIS sensitivity level at a binning of 8 and F/stop at 1. Missing petri dishes at 5 d.p.i. indicate animals that had succumbed to the infection or which were euthanized for ethical reasons. The colour code for the different analysed organs is indicated on the petri dish shown in (A). The colour bar indicates photon emission with 4 minutes integration time in photons/s/cm^2^/sr. Note, the red star in B indicates light signals emitted from a ruptured gallbladder accidentally punctuated during liver dissection. (**C**) Quantification of light emission signals shown in B at the indicated timepoints. Data represent means ± SEM, *p < 0.05; **p < 0.01; ***p < 0.001. Click here for file

Additional file 3: Figure S3 Survival of mice intragastrically inoculated with Lmo-EGD-lux or Lmo-InlA-mur-lux. Survival curves of female C57BL/6J, BALB/cJ, A/J OlaHsd, and C3HeB/FeJ mice inoculated intragastrically with 5 × 10^9^ CFU Lmo-EGD-lux (**A**) or Lmo-InlA-mur-lux (**B**). n = 10 for each mouse inbred and listerial strain. Click here for file
