# Supplementary figures and images for: Influence of internalin a murinisation on host resistance to orally acquired listeriosis in mice
Source: BMC Microbiol. 2013 Apr 23;13:90. doi: 10.1186/1471-2180-13-90 (PMC3640945; doi:10.1186/1471-2180-13-90)

**A**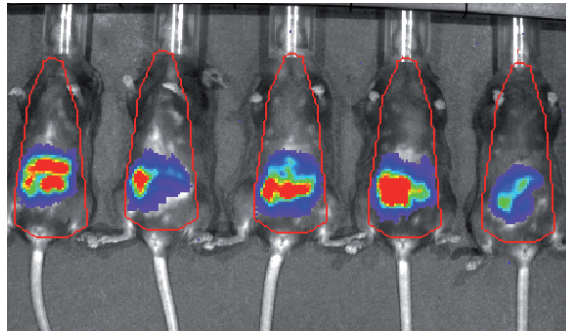**B****C3HeB/FeJ**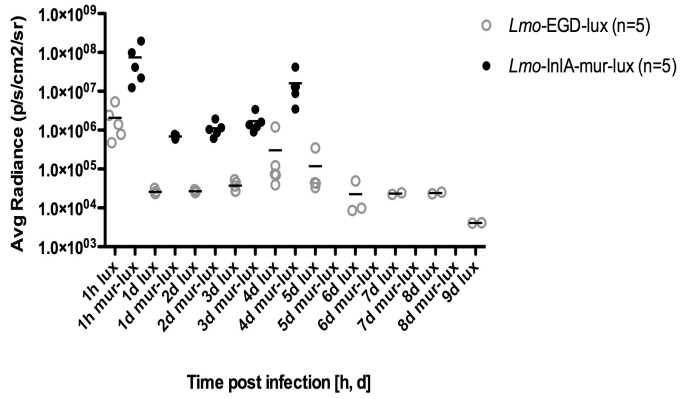**C****A/J OlaHsd**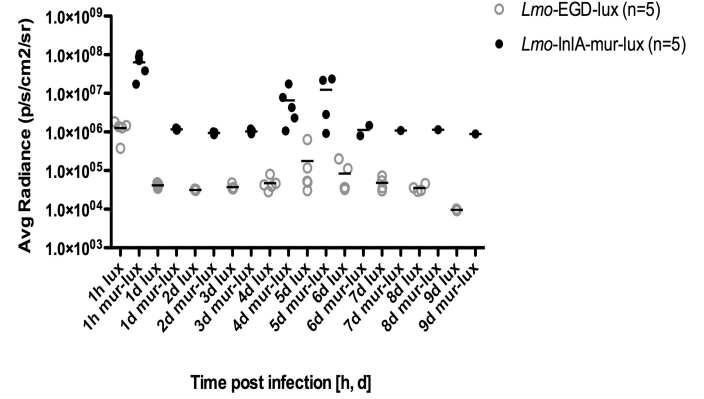**D****BALB/cJ**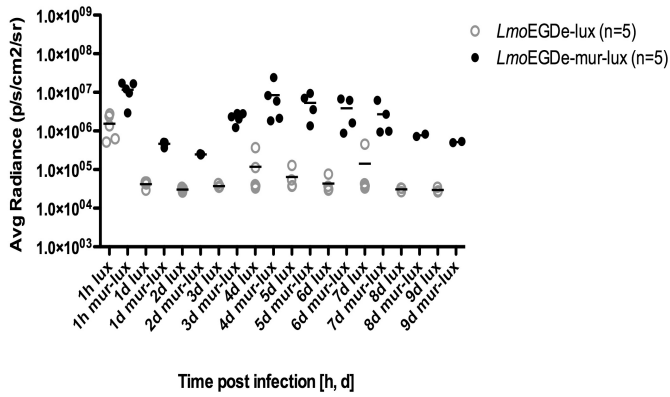**E****C57BL/6J**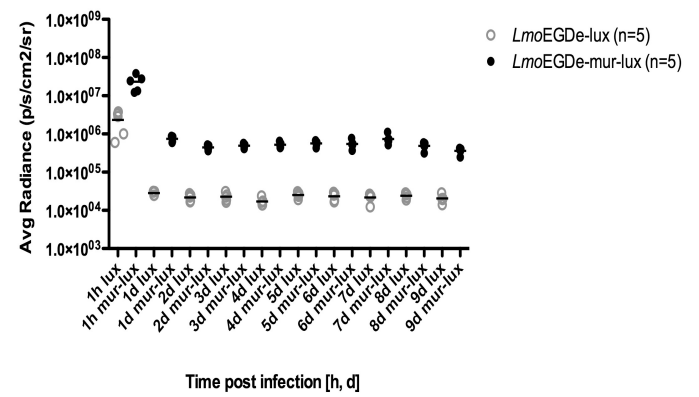

Supplement: Additional file 1: Figure S1 — Quantified BLI values from Figure 1. Light emission values from animals shown in Figure 1 were measured in an identical region in every mouse as shown in (A) and quantified as photons/s/cm2/sr. As described for Figure 1, mice from different inbred strains (n = 5, B-E) were intragastrically infected with 5 × 10^9^ CFU Lmo-EGD-lux (grey circles) or Lmo-InlA-mur-lux (black circles) and analysed for 9 days post infection. [file 1471-2180-13-90-S1.pdf]

**A**

Survival after *Lmo*-EGD-lux infection

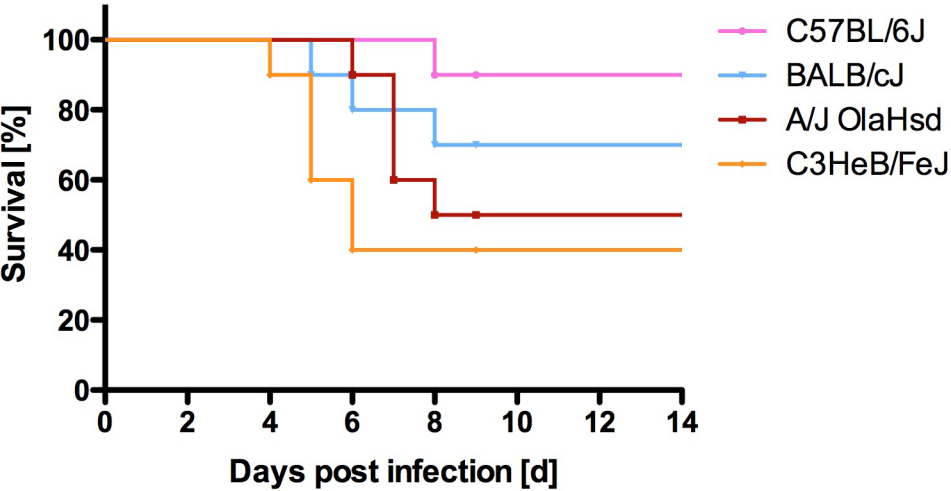**B**

Survival after *Lmo*-InIA-mur-lux infection

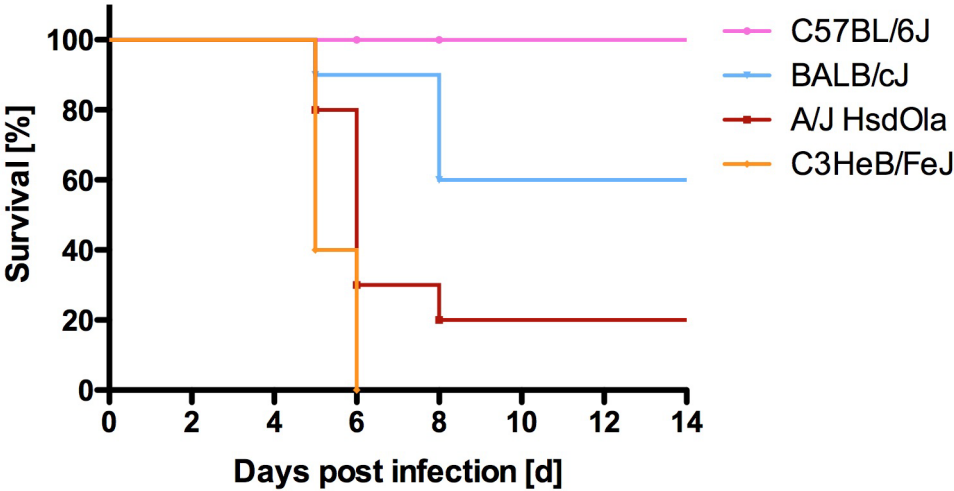

Supplement: Additional file 3: Figure S3 — Survival of mice intragastrically inoculated with Lmo-EGD-lux or Lmo-InlA-mur-lux. Survival curves of female C57BL/6J, BALB/cJ, A/J OlaHsd, and C3HeB/FeJ mice inoculated intragastrically with 5 × 10^9^ CFU Lmo-EGD-lux (A) or Lmo-InlA-mur-lux (B). n = 10 for each mouse inbred and listerial strain. [file 1471-2180-13-90-S3.pdf]
