# Supplementary material for: Influence of internalin a murinisation on host resistance to orally acquired listeriosis in mice
Source: BMC Microbiol. 2013 Apr 23;13:90. doi: 10.1186/1471-2180-13-90 (PMC3640945; doi:10.1186/1471-2180-13-90)
Supplement: Additional file 2: Figure S2 — Ex vivo BLI analysis of dissected internal organs. Six organs from Lmo-EGD-lux or Lmo-InlA-mur-lux infected animals (5 × 10^9^ CFU) were dissected at day 3 (3d) or day 5 (5d) post infection and imaged in an IVIS 200 imaging system. To aid interpretation of the figure a colour coded circle has been placed around each organ which emitted detectable light as shown in the example in (A). (B) Comparison of organ light emission signals in C3HeB/FeJ, A/J OlaHsd, BALB/cJ, and C57BL/6J female mice (n = 8, at day 0 of infection). The same imaging conditions were used for every organ by setting the IVIS sensitivity level at a binning of 8 and F/stop at 1. Missing petri dishes at 5 d.p.i. indicate animals that had succumbed to the infection or which were euthanized for ethical reasons. The colour code for the different analysed organs is indicated on the petri dish shown in (A). The colour bar indicates photon emission with 4 minutes integration time in photons/s/cm2/sr. Note, the red star in B indicates light signals emitted from a ruptured gallbladder accidentally punctuated during liver dissection. (C) Quantification of light emission signals shown in B at the indicated timepoints. Data represent means ± SEM, *p < 0.05; **p < 0.01; ***p < 0.001. [file 1471-2180-13-90-S2.pdf]

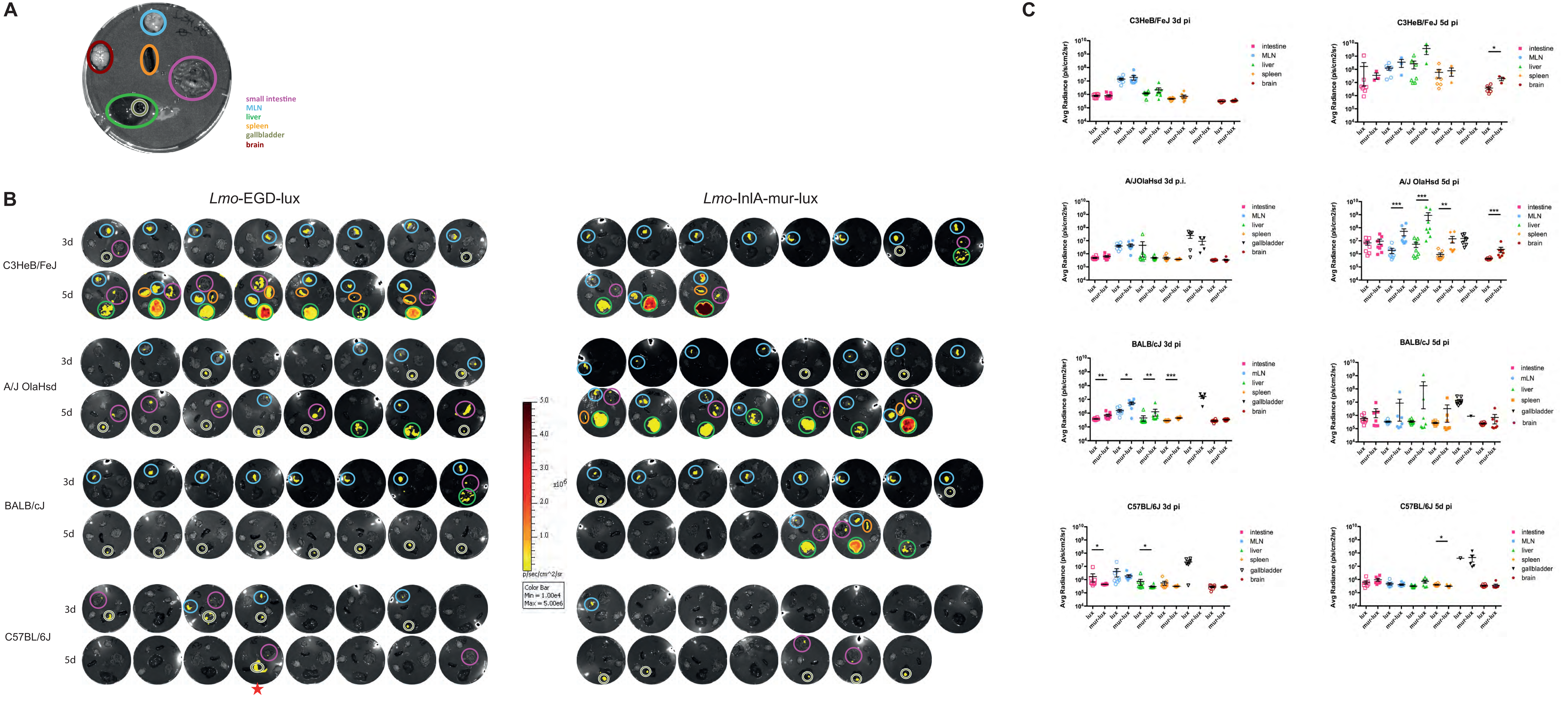

A
small intestine
MLN
liver
spleen
gallbladder
brain
B
Lmo-EGD-lux
Lmo-InlA-mur-lux
C3HeB/FeJ
A/J OlaHsd
BALB/cJ
C57BL/6J
3d
5d
p/sec/cm^2/sr
x10^6
Color Bar
Min = 1.00e4
Max = 5.00e6
C
C3HeB/FeJ 3d pi
C3HeB/FeJ 5d pi
A/JOlaHsd 3d p.i.
A/J OlaHsd 5d pi
BALB/cJ 3d pi
BALB/cJ 5d pi
C57BL/6J 3d pi
C57BL/6J 5d pi
Avg Radiance (p/s/cm2/sr)
lux
mur-lux
intestine
MLN
liver
spleen
gallbladder
brain
